# Structured chaos shapes joint spike-response noise entropy in temporally driven balanced networks

**DOI:** 10.1186/1471-2202-15-S1-P48

**Published:** 2014-07-21

**Authors:** Guillaume Lajoie, Jean-Philippe Thivierge, Eric Shea-Brown

**Affiliations:** 1 Department of Nonlinear Dynamics, Max Planck Institute for Dynamics and Self-Organization, Göttingen, 37018, Germany; 2 Department of Psychology, University of Ottawa, Ottawa, Ontario, Canada, K1N 6N5; 3 Department of Applied Mathematics, University of Washington, Seattle, Washington, 98195, USA

## 

How variable and noisy is the neural code arising from the joint activity of recurrently connected cells? Isolated neurons are known to respond to fluctuating input currents with reliable spike patterns [1,2], but variability in stimulus-evoked spike trains is increasingly pronounced in deeper, more recurrently connected brain areas such as cortex [3]. What are the network-level sources of this variability, and how they might constrain spiking features relevant for coding remains an open question.

We focus on spiking model networks with sparse, random connectivity and balanced excitation and inhibition that reproduce the irregular firing that typifies cortical activity. In such models, activity is known to be chaotic, with extremely strong sensitivity of spike outputs on tiny changes in a network’s initial conditions [4-6]. Nevertheless, when subject to temporally fluctuating driving inputs, networks can have chaotic attractors of limited dimension and geometric properties leading to reduced spiking variability at the single-cell level [7]. As recent studies suggest that the impact of noise on network coding cannot be understood by single cell properties alone [8,9], we study mechanisms underlying the joint activity of entire networks.

We derive a bound for the entropy of joint spike pattern distributions in large spiking model networks in response to a fluctuating temporal signal. The analysis is based on results from random dynamical systems theory and complimented by detailed numerical simulations. We find that despite very weak conditional correlations between neurons, the resulting joint variability of network responses is surprisingly lower than what would be expected by considering only limited statistical neural interactions. Moreover, joint spiking variability is strongly constrained by the level of temporal features of input stimuli.
